# Phenotypic and molecular characterization of *Staphylococcus aureus* from mobile phones in Nigeria

**DOI:** 10.3934/microbiol.2023021

**Published:** 2023-04-23

**Authors:** Anthonia O. Oluduro, Yetunde M. Adesiyan, Olumide O. Omoboye, Adebowale T. Odeyemi

**Affiliations:** 1 Department of Microbiology, Obafemi Awolowo University, Ile-Ife, 220005, Nigeria; 2 Landmark University SDG Groups 2 and 3; Department of Food Sciences and Microbiology, Landmark University, Omu-Aran, Kwara State, Nigeria

**Keywords:** mobile phone, *Staphylococcus aureus*, *nuc*, *mecA*, multiple antibiotic resistance

## Abstract

The presence of *Staphylococcus aureus*, a normal human flora on cellphones of different professionals in Ile-Ife was investigated with a view to determining their antibiotic susceptibility profile and nature of resistance and virulence genes. One hundred swab samples were collected aseptically from mobile phones of various users based on their profession. Surfaces of the mobile phones were swabbed and the streak plate method was used to isolate colonies showing characteristic golden yellow on mannitol salt agar plates. These isolates were further identified using standard microbiological methods. The antibiotic susceptibility of the isolates was determined using Kirby-Bauer's disk diffusion technique. Molecular detection of *nuc, mecA and pvl* genes in some isolates was carried out by polymerase chain reaction technique. All the 36 isolates obtained in this study were 100% resistant to amoxicillin and augmentin; the isolates also displayed 55.6%, 44.4% and 41.7% resistance to ceftriazone, erythromycin and chloramphenicol, respectively. Based on resistance to oxacillin, prevalence of methicillin resistant *Staphylococcus aureus* (MRSA) was 11.1%. Only one *S. aureus* was positive for plasmid analysis. *MecA* gene was genetically confirmed in four (4) out of the 16 suspected phenotypic MRSA strains, *nuc* gene was confirmed in all 28 isolates investigated, while there was no *pvl* gene in the strains investigated. Mobile phones harbor multiple antibiotics resistant *S. aureus, which* are responsible for important diseases in humans and could be difficult to manage with antibiotics thereby posing serious health risks.

## 1. Introduction

In Nigeria, there has been an increase in the use of mobile phones among the general population from more than forty million [1] to more than one hundred and eighty-four million in December 2019 [2].

They are handled frequently, and held to the face [3],[4] and this constant handling of the mobile phones by users makes it a breeding place for transmission of microorganisms as well as hospital-associated infections [4],[5]. The normal microbiota of the skin include among others Staphylococci which are also found regularly on clothes, bed linen and other human environments [6]. They can produce disease condition if introduced into foreign locations or compromised host [7]. It has been reported that Coagulase Negative *Staphylococcus* (CoNS), which constitute part of the normal flora of the skin is among the commonly isolated bacteria on cell phone and automated teller machine [8]–[11]. Also, potentially pathogenic bacteria commonly reported include methicillin sensitive *S. aureus* (MSSA), coliforms, methicillin resistant *S. aureus* (MRSA), *Corynebacterium* spp., *Enterococcus faecalis*, *Clostridium perfringens*, *Klebsiella* spp., *Enterobacter* spp., *Pseudomonas* spp., *Aeromonas* spp., *Acinetobacter* spp.*, Stenotrophomonas maltophilia*, *Escherichia coli, Klebsiella* spp. And *Bacillus* spp. [8],[9],[12]. *S. aureus* possess a number of virulent factors including cell surface factors, such as capsular polysaccharide; secreted factors for example, Panton-Valentine leucocidin (PVL), a typical cytolytic toxin, and various exoenzymes such as nucleases, proteases; among several others [13]. MRSA produce a transformed penicillin-binding protein (PBP), which is mediated by an acquired gene *mecA* and confers decreased affinity for most semisynthetic penicillins on the producing strains [14]–[17]. *MecA* is borne on a mobile genetic element (MGE) called staphylococcal cassette chromosome *mec* (SCC*mec*) [18],[19]. MRSA strains results from the horizontal transfer of the MGE by a competent strain(s), coupled with acquisition and insertion into the chromosomes of susceptible strains [20]. It has been reported that the acquisition of antimicrobial resistance poses a daunting task to the health sector with respect to the treatment and control of staphylococcal infections [20]. A study has shown that MRSA could account for up to 50% cases of *S. aureus* infections in hospitals [21]. MRSA infections are characterized with high morbidity and death rate which has been attributed to their resistance to all available penicillin and most of the other beta-lactam antibiotics, with the exception of ceftaroline and ceftobiprole [20]. Considering the health implications of mobile phones contaminated with methicillin resistant *S. aureus* viz a viz its economic cost, careful handling and observance of stringent hygiene is highly canvassed.

## 2. Materials and methods

### 2.1. Isolation of bacteria

The research was conducted at the Obafemi Awolowo University's Department of Microbiology Laboratory in Ile-Ife, Osun State, Nigeria. At Ile-Ife, Osun State, Nigeria, samples were taken from the mobile phones of different users based on their profession, including cleaners, abattoir employees, lecturers and students, nurses, food vendors and marketers.

All glassware used in this study was sterilized in the hot air oven at 160 °C for one hour. All the media used namely Mannitol Salt Agar (MSA), nutrient agar, nutrient broth and Mueller Hinton agar (MHA) were prepared according to the manufacturers' specifications. The reagents used were of analytical grade and were obtained from either Sigma Chemical Company or BDH.

The samples (n = 100) were collected aseptically by swabbing the surfaces of the keypad, mouthpiece and earpiece of the mobile phones using sterile cotton swab sticks moistened with sterile saline. The cotton swabs were then transported immediately to the laboratory for bacteriological analysis. The swabs were streaked onto Mannitol salt agar (MSA) plates and incubated aerobically at 37 °C for 24 to 48 hours. Colonies showing a characteristic golden yellow on MSA plates were picked and streaked on fresh MSA plates and incubated at 37 °C for 24 hours to obtain pure colonies, which were presumptively identified as *Staphylococcus aureus*. Preliminary characterization of isolates was carried out using Gram's staining and phenotypic characters, such as catalase, tube coagulase, nitrate reduction and DNase tests [22],[23].

### 2.2. Antibiotic susceptibility testing

Antibiotic susceptibility of isolates was determined on Mueller-Hinton agar plate by antibiotic disk diffusion method [24]. The antibiotics of known concentrations (Oxoid, USA); (amoxicillin (25 µg), ofloxacin (5 µg), streptomycin (30 µg), chloramphenicol (30 µg), ceftriaxone (30 µg), gentamicin (10 µg), pefloxacin (30 µg) cotrimoxazole (25 µg), ciprofloxacin (10 µg), erythromycin (5 µg), Augmentin (30 µg) and oxacillin (1 µg) was used. Standardized (0.05 MacFarland standard –10^8^ CFU/mL) *S. aureus* culture was seeded on cooled molten Mueller Hinton agar plate. The antibiotic disk was carefully placed on the seeded plate using sterile forceps, and incubated at 37 °C for 24 h. The diameter of zone of growth inhibition was measured in millimeter and recorded as resistant, susceptible or intermediate.

### 2.3. Molecular characterization of isolates

#### 2.3.1. Plasmid DNA extraction in *Staphylococcus aureus* isolates

Twenty-eight multiple antibiotic resistant isolates were randomly selected and profiled for plasmid analysis. The plasmid DNA of the *S. aureus* isolates were extracted using the modified alkaline lysis (TENS) method of Kraft *et al*. [25]. An overnight broth culture of the isolate grown in Luria-Bertani broth medium was spin in a 1.5 mL micro-centrifuge tube for 1 min to pellet the cells. The supernatant was gently decanted leaving 50–100 µL of the broth inside the tube. The tube was then vortexed at high speed to re-suspend the cells completely. After which 300 µL of TENS (Tris 25 Mm, EDTA 10 Mm, NaOH 0.1 N, and SDS 0.5%) was added to the tube and mixed by inverting the tubes 3–5 times until the mixture became sticky (caution was taken during this stage as stickiness ensure that lysis has taken place). After this, 150 mL of 3.0 M sodium acetate of pH 5.2 was added to the tube and vortexed to mix completely. After vortexing, the tube was spun in the micro-centrifuge for 5 min to pellet cell debris and chromosomal DNA. The supernatant was transferred into new tubes and mixed with 900 µL of ice-cold 100% ethanol. The tube was then centrifuged at 14,000 rpm for 10 min to pelletize the plasmid DNA. The supernatant was discarded leaving the whitish pellet in the tube. The whitish pellet was rinsed twice with 1 mL of 70% ethanol. The pellets were allowed to dry and were re-suspended in 20–40 µL of TE (Tris Ethylene diamine tetraacetate) buffer or distilled water for further use.

Purified plasmid DNA were then electrophoresed on 0.8% agarose gel stained with 1% ethidium bromide and ran at 80 V for 90 minutes for size estimation and pattern comparison with the marker (100 bp DNA ladder). The plasmid DNA bands were visualized by ultraviolet-trans-illuminator, photographed and documented using a gel electrophoresis documentation system. The molecular sizes of the unknown plasmid DNA were then estimated by comparing the distance travelled with that of the molecular weight of the standards.

### 2.4. Molecular characterization using polymerase chain reaction

#### 2.4.1. DNA extraction in *S. aureus* isolates

The DNA extraction was achieved through the microwave lysis method [26]. Twenty-eight (28) isolates were cultured in nutrient broth for 24 h at 37 °C with agitation at 100 rpm. The cell culture was centrifuged at 4,500 rpm for 5 min at 4 °C, the supernatant was poured off while the pellet obtained was washed with 1 mL of TE (10 mM Tris, pH 8, 10 mM EDTA) and was re-suspended in 100 µL of TE. Fifty microliters (50 µL) of 10% SDS was added to the mixture and incubated for 30 min at 65 °C. The lysate was centrifuged and supernatants removed. The micro-tubes were then placed in a microwave oven (with specification; LG grill, model No. MG-604AZ, input 220v–50/bHz, microwave 1350 W, RF output 900 W, 2450 MH) and heated twice for 1 min at 900 W or three times for 1 min at 750 W. Each pellet was dissolved in 200 µL of TE and were extracted with an equal volume of phenol/chloroform/isoamyl alcohol (25:24:1) for 15 min. The aqueous phase was recovered by centrifugation for 20 min and precipitated with ethanol and kept in the refrigerator at 4 °C until needed for polymerase chain reaction (PCR) amplification.

#### 2.4.2. Molecular detection of thermonuclease (nuc) and mecA genes by polymerase chain reaction

Twenty eight (28) multiple antibiotic resistant *Staphylococcus aureus* isolates were selected based on their resistance profile and screened for the presence of thermonuclease (*nuc*) and *mecA* genes. Primers which amplified a 279 bp segment of the *nuc* gene and a 147 bp segment of the *mecA* gene were used for this study (Table 1). All PCR reactions were carried out separately in a final volume of 25 µL of a PCR reaction mixture containing 1 µL MgCl_2,_ 5 µL of the master mix consisting of dNTPs and Taq polymerase, 1 µL of each primer (both reverse and forward), and 7 µL of the template DNA with 10 µL of nuclease-free water. The thermocycler was programmed with the following parameters for the amplification of *nuc* gene: an initial denaturation at 95 °C for 2 minutes, 40 cycles of denaturation at 95 °C for 30 seconds, annealing temperature of 54 °C for 30 seconds, elongation at 72 °C for 45 seconds and final extension at 72 °C for 5 minutes [27].

For the amplification of *mecA* gene, the thermocycler was programmed with the following parameters: an initial denaturation at 95 °C for 3 minutes, 30 cycles denaturation at 95 °C for 30 seconds, annealing temperature of 60 °C for 30 seconds, elongation at 72 °C for 30 seconds and final extension at 72 °C for 10 minutes [28]. The PCR products were analyzed by gel electrophoresis using 1% agarose using IxTBE buffer (0.089 M boric acid, 0.002 M EDTA disodium buffer, pH 8.3) for 1 hour at 100 V. The gel was visualized under ultraviolet light and analyzed by comparing the corresponding band for the specific gene with the positive control and DNA ladder.

#### 2.4.3. Molecular detection of *pvl* genes in *Staphylococcus aureus* isolates

A 25 µL of the PCR reaction mixture contains 1 µL MgCl_2_ 4 µL of PCR buffer, 3 µL of each dNTP, 0.2 µL of Taq polymerase, 1 µL of each primer (reverse and forward), 9.8 µL nuclease-free water and 5 µL of the DNA sample. The thermo cycler (Eppendorf Mastercycler, USA) was programmed for optimum conditions. The PCR mixture was poured into PCR tubes and vortexed for proper mixing before loading them into the thermocycler. The PCR amplification condition of the thermocycler was set as follows: an initial denaturation at 95 °C for 5 min, 35 cycles of denaturation at 95 °C for 1 minutes, annealing temperature of 65 °C for 1 min, elongation at 72 °C for 1 min and final extension at 72 °C for 10 min [29]. Gel electrophoresis was used to detect the amplified DNA products. A volume of 10 µL of the amplified PCR products was subjected to electrophoresis at 100 volts for about 2 hours. The marker used was a 100 bp ladder, which was added to one of the wells in the gel to serve as reference. The amplified product was viewed using an Ultraviolet transilluminator. The Primers used for the molecular detection of *pvl* gene is presented in Table 1.

**Table 1.** Primers used for the molecular detection of *nuc, mecA* and *pvl* genes.

Gene		Primers	References
Thermonuclease (*nuc* gene) 279 bp	Forward *nuc1*	5′GCGATTGATGGTGATACGGTT3′	Brakstad *et al*., 1992
	Reverse *nuc2*	5′AGCCAAGCCTTGACGAACTAAAGC3′	
Methicillin resistant *mecA* gene (147 bp)	Forward *mec1*	5′GTGAAGATATACCAAGTGATT3′	Zhang *et al*., 2005
	Reverse *mec2*	5′ATGCGCTATAGATTGAAAGGAT3′	
Panton Valentine Leucocidine *pvl* gene (433 bp)	Forward *pvl1*	5′ATCATTAGGTAAAATGTCTGGACATGATCCA3′	Lina *et al*., 1999
	Reverse *pvl2*	5′GCATCAAGTGTATTGGATAGCAAAAGC3′	

## 3. Results

### 3.1. Social and demographic characteristics of study subjects

A total of 100 (32 male and 68 female) persons from 5 different professional backgrounds (nurses, students/lecturers, abattoir workers, food vendors/market women and cleaners) in Ile-Ife (OAU Campus, OAUTHC, Opa area and Odo-Ogbe Market) participated in this study. Their ages ranged between 17 and 58 years and their mean (±SE) age was 35.99 (±0.89) years with majority (46%) in the age range of 27–36 years. They've had phones for no less than three months or more than 144 months. Most of them (54.6%) have been mobile phone users for between one and five years. Table 2 lists participant responses to questions on cell phone usage. The majority of participants use the same mobile phones at work and at home, share them with coworkers and don't frequently clean them.

**Table 2.** Socio-demographic characteristics (sex) of mobile phone users in relation to hygienic habits during usage.

	Users attributes/characteristics	No of Samples (N = 100)	Phone usage at workplace		Phone usage at home		Usage of same phone at home and at workplace		Answering of phone calls while working		Wearing of glove/ protective covering while answering phone calls		phone cleaning in the past		Regular phone cleaning		Regular hand washing after phone use		Receiving/making of phone calls/ texting while toileting		Knowledge of transfer of pathogenic bacteria via phone usage	
			Yes	No	Yes	No	Yes	No	Yes	No	Yes	No	Yes	No	Yes	No	Yes	No	Yes	No	Yes	No
Sex	Male	32	28	4	32	0	30	2	27	5	6	26	2	30	1	31	0	32	23	9	3	29
	Female	68	62	6	68	0	64	4	52	16	24	44	7	61	3	65	0	68	52	16	12	56
	X^2^		0.05*		75.92		0.144*		0.82		2.84		0.081*		0.058*		75.92		0.245		0.609	
	P		0.831*		0		0.704		0.365		0.092		0.776*		0.810*		0		0.621		0.435	
Age	17–26	13	13	0	13	0	13	0	12	1	2	11	0	13	0	13	0	13	9	4	1	12
	27–36	46	43	3	46	0	43	3	39	7	13	33	3	43	2	44	0	46	40	6	10	36
	37–46	26	23	3	26	0	23	2	21	5	4	22	2	24	1	14	0	26	23	3	3	23
	47–56	15	13	4	15	0	14	1	7	8	5	10	4	11	1	14	0	15	3	12	1	14
	X^2^		5.684		104.88		1.82		11.816		2.761		7.401		0.836		104.88		30.451		3.244	
	P		0.128		0		0.611		0.008		0.42996		0.06016		0.84084		0		0.0000011		0.3555	
Occupation	Student/Lecturer	20	19	1	20	0	18	2	16	4	3	17	1	19	1	19	0	20	14	6	4	16
	Nurses	20	17	3	20	0	16	4	12	8	6	14	4	16	3	17	0	20	16	4	10	10
	Food Vendors/Market Women	20	19	1	20	0	20	0	19	1	4	16	1	19	0	20	0	20	15	5	0	20
	Abattoir Workers/Butchers	20	17	3	20	0	20	0	16	4	3	17	1	19	0	20	0	20	15	5	0	20
Occupation	Cleaners	20	18	2	20	0	20	0	16	4	8	12	2	18	0	20	0	20	15	5	1	19
	X^2^		2.222		50		4.756		7.478		5.154		4.151		4.232		50		0.533		22.451	
	P		0.695		1.10E-07		0.313		0.113		0.272		0.386		0.376		1.10E-07		0.97		0.000163	

*Yates correction for continuity (Preacher, 2001) was employed to improve the accuracy of the null-condition sampling distribution of chi-square.

**Table 3.** Distribution of *S. aureus*, MRSA and MSSA isolates from mobile phones from mobile phones of the investigated users.

Categories of users	No of *S. aureus* (%)	Number of MRSA (%)	Number of MSSA (%)
Students/Lecturers	13 (36.1)	2 (12.5)	11 (55)
Nurses	1 (2.8)	0	1 (5)
Abattoir workers/Butchers	4 (11.1)	8 (50)	6 (30)
Food Vendors/Market Women	14 (38.9)	3 (18.8)	1 (5)
Cleaners	4 (11.1)	3 (18.8)	1 (5)
Total	36 (100)	16	20

T-test of MRSA and MSSA isolates from investigated users' mobile phone at p < 0.05. t_α0.05_ -0.334, P = 0.747.

Virtually all the respondents irrespective of their gender, age and occupation neither wash their hands after using mobile phones nor before attending to customers/patients nor shake other people's hands. The majority of the respondents answer/receive phone calls, text/send short message service (sms) and/or other purposes (e.g., lighting, browsing and so on) while in the toilet. Meanwhile 50% of the nurses and 20% of students/lecturers believe that mobile phones can serve as a medium of transferring bacteria from person to person (Table 2). All responses, when statistically tested for independence using Chi-square at p < 0.005, were independent of the respondents' gender, age and profession except ‘answering of phone calls while working’ and ‘receiving/making of phone calls/texting while in the rest room which are age-dependent; and ‘knowledge of transfer of pathogenic bacteria via phone usage’ which is profession-dependent. In other words, most of the respondents' answers relating to hygienic use of mobile phones are not affected or determined by their (respondents) socio-demographic statistics.

The result presented in Table 3, also revealed that all the mobile phones of the investigated categories of users were contaminated with *Staphylococcus aureus*. The mobile phones of food vendors/market women (38.89%) and students/lecturers (36.11%) had the highest level of *S. aureus* contamination, followed by that of the abattoir workers/butchers (11.11%) and cleaners (11.11%), while that of Nurses were the least contaminated (2.78%).

### 3.2. Methicillin resistant and methicillin sensitive *S. aureus* isolated from mobile phones of investigated users

Isolates were considered methicillin resistant or sensitive based on its resistance or sensitivity to oxacillin. Out of the 36 isolated *S. aureus* from mobile phones of investigated users, 16 (44.44%) were discovered to be methicillin resistant *S. aureus* (MRSA) and the other 20 (55.56%) were methicilin sensitive *S. aureus* (MSSA) (Table 3). Out of the sixteen MRSA detected, 8 (50%) were recovered from food vendors/market women mobile phones, Abattoir workers/Butcher 3 (18.75%), Cleaner 3 (18.75), Students/lecturers 2 (12.50%). The number of MRSA is not significant different from MSSA (Table 3).

### 3.3. Isolation and identification of *Staphylococcus aureus* isolates

A total of 115 *Staphylococcus* sp. were recovered from 100 mobile phones of various users out of which 36 *Staphylococcus aureus* were identified based on their cultural, morphological and biochemical characteristics. Based on Gram's staining, the isolates were found to be Gram positive cocci. The result of the cultural characteristics of the isolates showed that their colonies were golden yellow when cultured on mannitol salt agar (MSA). The results of the morphological characteristics showed that they were Gram positive, and clustered cocci. The isolates were positive for tube coagulase test, catalase, DNAse and nitrate reduction test.

### 3.4. Antibiotic susceptibility and multiple antibiotic resistance patterns of the *S. aureus* isolates

All the 36 *S. aureus* isolates recovered from the cellphones of the investigated users were resistant to amoxicillin and augmentin. Also, 55.6%, 44.4% and 41.7% of the strains were resistant to cefriazone, erythromycin and chloramphenicol, respectively (Table 4). In addition, 12 (33.33%) of the 36 *Staphylococcus aureus* isolates displayed multiple antibiotic resistance patterns to the antibiotics (three to six different classes) assessed in this study as shown in Table 5. There was diversity in multiple antibiotic resistance (MAR) patterns; 5 (13.9%) isolates displayed MAR to 3 and 4 classes of antibiotics, respectively, while 1 (2.8%) isolate was resistant to 5 and 6 classes of antibiotics, respectively. The predominant MAR phenotype was “AMX, STR, CHL, ERY” which was observed in 5 (13.9%) of the isolates.

**Table 4.** Frequency of antibiotic resistance and susceptibility of *S. aureus* isolates from mobile phones.

Antibiotics	Frequency (%)		
	Resistance	Intermediate	Susceptible
Amoxicillin (25 µg)	36 (100)	0	0
Ofloxacin (10 µg)	0	1 (2.8)	35 (97.2)
Streptomycin (30 µg)	8 (22.2)	0	28 (77.8)
Chloramphenicol (30 µg)	15 (41.7)	1 (2.8)	20 (55.6)
Ceftriazone (30 µg)	20 (55.6)	9 (25)	7 (19.4)
Gentamycin (10 µg)	6 (16.7)	1 (2.8)	29 (80.6)
Pefloxacin (30 µg)	0	1 (2.8)	35 (97.2)
Cotrimoxazole (25 µg)	4 (11.1)	0	32 (88.9)
Ciprofloxacin (10 µg)	1 (2.8)	7 (19.4)	28 (77.8)
Erythromycin (5 µg)	16 (44.4)	11 (30.6)	9 (25)
Augmentin (30 µg)	36 (100)	0	0
Oxacillin (1 µg)	15 (41.7)	3 (8.3)	18 (50)

**Table 5.** Multiple antibiotic resistance patterns of the *S. aureus* isolates from mobile phones.

Number of classes of antibiotics	Multiple antibiotic resistance pattern	Number of isolates	Total (%)
3	AMX, CHL, ERY	3	5 (13.9)
	AMX, STR, CHL	1	
	AMX, COT, ERY	1	
4	AMX, STR, CHL, ERY	5	5(13.9)
5	AMX, STR, CHL, COT, ERY	1	1 (2.8)
6	AMX, STR, CHL, COT, CIP, ERY	1	1 (2.8)
	Sum total	12	12 (33.33)

*Total number of isolate: 36, AMX: Amoxicillin, STR: Streptomycin, CHL: Chloramphenicol, COT: Cotrimoxazole, ERY: Erythromycin, CIP: Ciprofloxacin

### 3.5. Plasmid DNA detection in selected multiple antibiotic resistant *Staphylococcus aureus*

**Figure 1.**
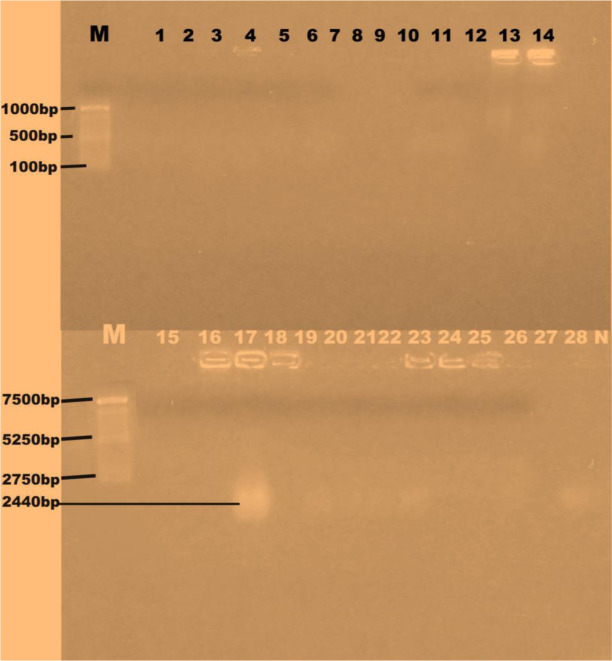
Agarose gel electrophoresis of plasmids in multiple antibiotic resistant *Staphylococcus aureus* isolates. Lane M=DNA marker (HIND III digest), Lanes 1–14, 15–16, 18–28 have no plasmids while Lane 17 is positive for plasmid (2440 bp). Lane N = Negative control, Lanes 1–7 = Students/Lecturers, Lanes 8–10 = Cleaners, Lanes 11–12 = Abattoir workers/ butchers, Lanes 13–28 = Food vendors/market women. *L17: *S. aureus* isolate from food vendor mobile phone.

Twenty-eight *S. aureus* isolates were selected for plasmid analysis. Only one isolate possessed plasmid DNA of molecular weight of 2440 bp. Figure 1 shows the result of the agarose gel electrophoresis of plasmids in the multiple antibiotic resistant *Staphylococcus aureus* isolates.

### 3.6. Molecular detection of *nuc, mecA* and *pvl* genes

**Figure 2.**
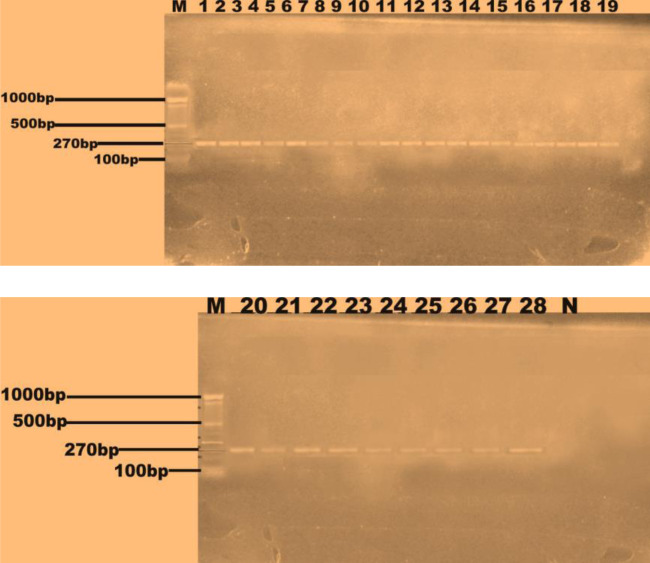
PCR amplification of *nuc* gene (270 bp) in *Staphylococcus aureus* isolates. Lane M = 100 bp marker, Lane N = Negative control, Lanes 1–7: Students/lecturers, Lanes 8–10: Cleaners, Lanes 11–12: Abattoir workers/butchers, Lanes 13–28: Food vendors/market women.

**Figure 3.**
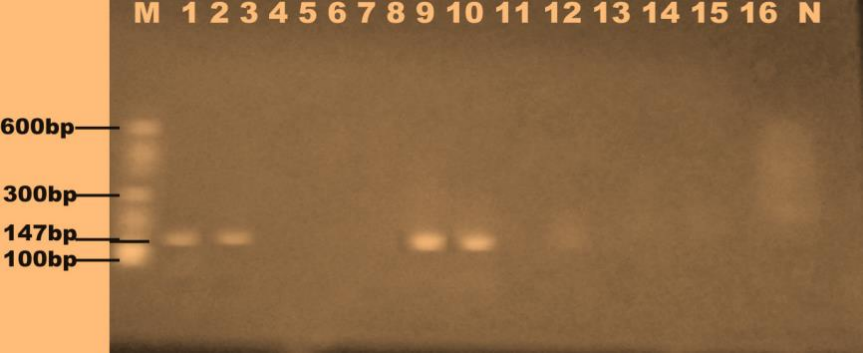
PCR amplification of *mecA* (147 bp) gene in *Staphylococcus aureus* isolates. M=100 bp marker, Lane N = Negative control, Lanes 1, 2, 9, 10, 12–16 = Food vendors/market women, Lanes 3–5 = Students/lecturers, Lanes 6–8 = Cleaners, Lane 11 = Abattoir workers/butchers.

**Figure 4.**
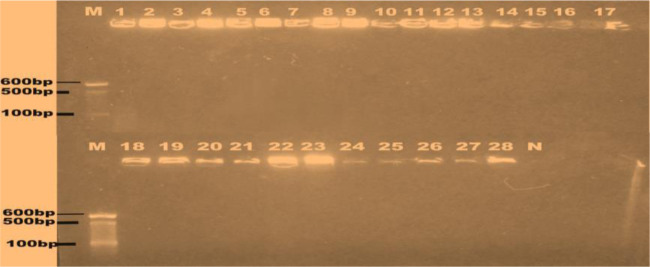
PCR amplification showing the absence of *pvl* gene in the *Staphylococcus aureus* isolates. Lane M=100 bp marker, Lane N = Negative control, Lanes 1–7 = Students/lecturers, Lanes 8–10 = Cleaners, Lanes 11–12 = Abattoir workers/butchers, Lanes 13–28 = Food vendors/market women.

Twenty eight multiple antibiotic resistant *S. aureus* isolates were selected based on the results of the antibiotic susceptibility profiles. *Nuc* (279 bp) gene was detected in all the twenty-eight isolates (Figure 2), while *mecA* (147 bp) gene was detected in four out of the sixteen isolates recovered from the food vendors that were phenotypically resistant to methicillin as depicted by lanes 1, 2, 9 and 10 in Figure 3. *Pvl* gene was not detected in all the *S. aureus* isolates from mobile phones as shown in Figure 4. The NanoDrop-1000 spectrophotometer was used to determine the subject's DNA's purity, and the appropriate yields of 1.13 (the lowest) to 149 ng/L (the highest) were achieved at 260 and 280 nm. The absorbance ratio was compared. As a result, highly pure DNA products were obtained, and PCR results revealed that each one had the correct gene that codes for the appropriate drug-metabolizing enzyme. Both the quantity and purity of the DNA in the samples may be measured with the help of the NanoDrop-1000 method and PCR analysis.

## 4. Discussion

Mobile phones are reservoirs for pathogenic bacteria owing to its unhygienic handling [30], could serves as vehicle the transmission of hospital acquired pathogens including MRSA [31]. In this study, we investigated the multiple antibiotic resistance and profiled virulence genes associated with *S. aureus* isolated from mobile phones of different professionals in Ile-Ife, Osun State, Nigeria.

The isolation of *S. aureus* from mobile phones of the investigated users is in agreement with the reports of several researchers who had reported the occurrence of *S. aureus* on mobile phones [7],[32]–[35]. Although *S. aureus* is a normal flora of the skin, it can cause serious health challenges and complications if it has access into the body such as wounds and the blood stream. High percentage of *S. aureus* isolated from mobile phones of food vendors/market women and students in this study could be attributed to the dynamic nature of these two sets of people. Vendors/market women interact with a lot of people in the course of transaction with involves the exchange of materials for money, while students interact with one in the lecture rooms, halls of residents, university campus and community at large; another through handshakes, hugging, sharing of phones, writing materials and clothing. These activities could promote the transmission of *S. aureus*. Isolation of *S. aureus* from mobile phones and various computer interphase devices in communities worldwide have been reported by previous researchers [11],[34],[36]–[39]. Ilusanya *et al*. [37] reported that *S. aureus* accounted for 50% of the isolates obtained from the mobile phones of food vendors, while Roy *et al*. [38] showed that *S. aureus* was 12% of bacteria obtained from mobile phones of students. In addition, Adhikari *et al*. [40] reported 56% incidence of *S. aureus* on the phones of staff and students of Birendra Multiple Campus, Bharatpur in Nepal; while Ulger *et al*. [41], Elmanama *et al*. [42] and Gashaw *et al*. [43] reported 52%, 27% and 21%, in Turkey, Palestine and Ethiopia, respectively. Interestingly, the least percentage of *S. aureus* isolated from the mobile phones of nurses in this study is similar to the findings of Famurewa and David [36] who reported low occurrence of the bacteria in their study. The low incidence of the bacteria on the mobile phones of nurses may be because they are aware that inanimate objects and surfaces at the hospitals could easily transmit *S. aureus*. This could have contributed to possible precautionary measures taken in the course of their job, such as wearing of gloves and other protective clothing, washing of hands, hygienic handling of materials and surfaces at the hospital settings and disinfection of their hands before handling their mobile phones and other portable computers.

The presence of MRSA on mobile phones of student/lecturer, abattoir workers, cleaners and most especially, those of the food vendors/market women is worrisome. This can predispose the respondents and other individuals in the community to increasing exposure to community-acquired MRSA infections, which is fast evolving resistance to commonly used antibiotics [44]. It is known that MRSA synthesize an altered penicillin-binding protein (PBP) encoded by *mecA* which is responsible for the decreased affinity for majority of semisynthetic penicillins (as reviewed by Lakhundi and Zhang [20].

Wangai *et al*. [45] reported that the detection of *S. aureus*-specific gene markers like *nuc* [46] could be used to delineate MRSA from coagulase negative *S. aureus* which also harbour *mecA* gene. Interestingly, all the *S. aureus* in this study harbored *nuc* gene confirming them as *S. aureus* while 25% of the 16 phenotypic MRSA was confirmed genetically by the presence of *mecA* gene. We observed 33.3% MAR *S. aureus* and 44.4% MRSA in this study. However, Adhirikai *et al*. [40] reported 21.4% MAR *S. aureus* and 26.8% MRSA. MRSA was initially described solely as nosocomial infection-causing bacteria, but it has been reported that the bacteria could cause community-acquired infections [20]. It has been reported that infections due to MRSA is responsible for high death rate, prolonged hospital stay and higher health care costs in comparison to infections caused by MSSA [47]–[50]. Isolation of high percentage of MAR *S. aureus* and MRSA from the mobile phones of several professionals at Ile-Ife, Nigeria is a cause for concern because it could trigger a disease outbreak if this observation is not properly handled. It was observed in this study that only one representative of the multiple antibiotic resistant (MAR) *S. aureus* was found to harbor class1 plasmid which indicates the range of 1–5 kb. The absence of plasmid in most of the MAR isolates could be that their antibiotic resistance is not plasmids-borne but chromosomal. The chromosomal-borne antibiotic resistance observed in this study could minimize the dissemination of antibiotic resistance gene among bacteria associated with the microflora of the respondents, in contrast to plasmids-borne antibiotic resistance gene that can be rapidly spread through horizontal gene transfer. PCR and the NanoDrop-1000 spectrophotometer are two extremely precise techniques for measuring genomic DNA. DNA quality has a direct impact on the effectiveness of Polymerase Chain Reaction amplification. The amount of genes present that code for drug-metabolizing enzymes increases with purity, amplification efficiency and gene quantity [51].

## 5. Conclusion

Hygienic use of mobile phones is independent of sex, age and occupation/profession. However, the observation that many health workers in line of duty are inseparable from their mobile phones while attending to patients is worrisome. The isolation of multiple antibiotic resistant *S. aureus* and MRSA from the mobile phones of several professionals in Ile-Ife is an insight to possible disease outbreak form this pathogen which requires proper surveillance and awareness to keep the community safe. Antibiotic-resistant bacteria might be transmitted using cell phones and portable computers. In order to reduce the local and global transmission of bacterial resistant strains, frequent hand washing and surface disinfection of mobile phones and portable computers are advised everywhere. Aggressive awareness on the need for hygienic handling of mobile phones and handy computer devices by all and sundry, especially health workers is highly recommended.
